# Comprehensive proteome profiling in *Aedes albopictus* to decipher *Wolbachia-*arbovirus interference phenomenon

**DOI:** 10.1186/s12864-017-3985-y

**Published:** 2017-08-18

**Authors:** Yoann Saucereau, Claire Valiente Moro, Cindy Dieryckx, Jean-William Dupuy, Florence-Hélène Tran, Vincent Girard, Patrick Potier, Patrick Mavingui

**Affiliations:** 1 Université de Lyon, Lyon, France; 2 Université Lyon 1, Villeurbanne, France; 3 CNRS, UMR5557, Ecologie Microbienne, Villeurbanne, France; 4 INRA, UMR1418, Villeurbanne, France; 5 Laboratoire Mixte UMR 5240, Plateforme de Protéomique, CNRS, Lyon, France; 6 Centre de Génomique Fonctionnelle, Plateforme Protéome, Université Bordeaux, F-33000 Bordeaux, France; 7 CNRS 9192, INSERM U1187, IRD 249, Unité Mixte Processus Infectieux en Milieu Insulaire Tropical (PIMIT). Plateforme Technologique CYROI, Université de La Réunion, 2 rue Maxime Rivière, 97490 Sainte Clotilde, île de La Réunion France

**Keywords:** *Aedes albopictus*, *Wolbachia*, Viral inhibition, Proteome, Glycolysis, Metabolism, miRNA

## Abstract

**Background:**

*Aedes albopictus* is a vector of arboviruses that cause severe diseases in humans such as Chikungunya, Dengue and Zika fevers. The vector competence of *Ae. albopictus* varies depending on the mosquito population involved and the virus transmitted. *Wolbachia* infection status in believed to be among key elements that determine viral transmission efficiency. Little is known about the cellular functions mobilized in *Ae. albopictus* during co-infection by *Wolbachia* and a given arbovirus. To decipher this tripartite interaction at the molecular level, we performed a proteome analysis in *Ae. albopictus* C6/36 cells mono-infected by *Wolbachia w*AlbB strain or Chikungunya virus (CHIKV), and bi-infected.

**Results:**

We first confirmed significant inhibition of CHIKV by *Wolbachia*. Using two-dimensional gel electrophoresis followed by nano liquid chromatography coupled with tandem mass spectrometry, we identified 600 unique differentially expressed proteins mostly related to glycolysis, translation and protein metabolism. *Wolbachia* infection had greater impact on cellular functions than CHIKV infection, inducing either up or down-regulation of proteins associated with metabolic processes such as glycolysis and ATP metabolism, or structural glycoproteins and capsid proteins in the case of bi-infection with CHIKV. CHIKV infection inhibited expression of proteins linked with the processes of transcription, translation, lipid storage and miRNA pathways.

**Conclusions:**

The results of our proteome profiling have provided new insights into the molecular pathways involved in tripartite *Ae. albopictus*-*Wolbachia*-CHIKV interaction and may help defining targets for the better implementation of *Wolbachia*-based strategies for disease transmission control.

**Electronic supplementary material:**

The online version of this article (doi:10.1186/s12864-017-3985-y) contains supplementary material, which is available to authorized users.

## Background

The Asian tiger mosquito *Aedes albopictus* is a species native to South and East Asia, with a great capacity for invasion. It has been classified by the WHO as the fourth most invasive species in the world [1]. Since the mid-twentieth century, *Ae. albopictus* has considerably increased its distribution, and is currently present on five continents [2]. *Ae. albopictus* is involved in the transmission of many human-infecting arboviruses, including Chikungunya virus (CHIKV), Dengue virus (DENV), and probably Zika virus [3–5]. Historically, *Ae. Albopictus* has been considered of secondary importance in terms of arbovirosis incidence relative to *Aedes aegypti*. However, this has changed since the implication of *Ae. albopictus* in the explosive epidemics of CHIKV on La Reunion Island and neighboring islands in southern Indian Ocean [6, 7], as well as in the CHIKV outbreaks in Italy [8] and successive autochthonous transmissions of both CHIKV and DENV in metropolitan France [9–12]. Efficient transmission of CHIKV has been associated with a mutation in E1 envelope glycoprotein (Ala-226-Val) that increases viral infectivity in *Ae. albopictus* compared to *Ae. aegypti* [6, 13]. Advances in technologies of large-scale analysis and the availability of genome sequencing allow the meaning of this host tolerance to be examined at the molecular level by the screening of cell factors possibly mobilized during viral cell invasion. In *Ae. aegypti* differential trends of proteomic expression were seen in the midgut and salivary glands infected by CHIKV or DENV in comparison to uninfected specimens [14, 15]. Using cellular models, microarrays studies have shown that CHIKV enters *Ae. albopictus* cells by clathrin-dependent endocytosis [16], activating diverse biological processes, including protein folding and metabolic pathways [17]. Overall, the modulation of the synthesis of some classes of host proteins clearly favors virus survival, replication and transmission [18].

*Ae. albopictus* is naturally infected by the intracellular bacterium *Wolbachia pipientis* that are maternally transmitted from mother to offspring. Two distinct *Wolbachia* strains (*w*AlbA and *w*AlbB) are present in variable density in *Ae. albopictus* tissues [19–21] and they usually induce sterility through the phenomenon known as cytoplasmic incompatibility [22–24]. In *Ae. aegypti*, naturally devoid of *Wolbachia*, transinfected females harboring the *w*AlbB strain have been found to inhibit the transmission of both CHIKV and DENV [25, 26]. In *Ae. albopictus* dissemination of DENV serotype 2 to salivary glands of *Wolbachia*-infected *Ae. albopictus* from La Reunion was considerably diminished in comparison to *Wolbachia*-uninfected individuals generated by antibiotic treatment [27]. When *Ae. albopictus* was transinfected with *Wolbachia w*Mel strain derived from *Drosophila melanogaster,* the transmission of DENV serotype 2 was totally abolished [28]. However, the inhibitory effect of *Wolbachia* is not universal [29, 30], and one study noted an increase in parasite infection in *Anopheles* [31], suggesting that variable mechanisms are involved depending on the interacting partners. Investigations into the molecular mechanisms behind *Wolbachia* interference have suggested that the bacterium may act by modulating expression of insect innate immune genes, including antimicrobial peptides, or more broadly by inducing oxidative and metabolic stresses that will in turn impact the behavior of the infectious agent in the host cells [32, 33]. It is also proposed that *Wolbachia* and viruses would compete for the host cells’ resources [34].

We recently showed that the *w*AlbB strain was able to block CHIKV infection in *Ae. albopictus* C6/36 cell lines relative to uninfected cells [35]. This is in line with observations in all studies using cellular models [36, 37], suggesting that viral inhibition is common in such simplified systems, possibly due in part to the proximity of the interacting partners. Thus, cellular models could represent interesting systems to decipher the mechanisms involved in the tripartite interactions between *Wolbachia*, arboviruses and host cells. Both naturally and artificially *Wolbachia*-infected *Aedes* cell lines have shown changes in the expression of several genes involved in structural, metabolic and stress functions [38, 39]. On the other hand CHIKV was reported to activate cellular functions necessary for infection and persistence [17]. However, no molecular mechanism for the interplay between *Wolbachia* and CHIKV in *Ae. albopictus* has been proposed to date. Therefore, in this study we used proteome profiling of *Ae. albopictus* C6/36 cell lines to discover how *Wolbachia*-infected cells reacted when challenged with CHIKV. Two-dimensional electrophoresis (2DE) followed by nano liquid chromatography and coupled with tandem mass spectrometry (nanoLC-MS/MS) showed differentially expressed proteins likely belonging to diverse processes of glycolysis, protein metabolism, protein modification and amino acid metabolism. Overall, the innovative proteomic approach used in this descriptive work provided potential candidates involved in the tripartite interaction between mosquito-CHIKV-*Wolbachia*. Future investigation will focus on the functional studies to validate the more promising candidates implicated in cellular processes that mediated the interplay between microbes.

## Methods

### Mosquito cell line and virus

The C6/36 cells infected by *Wolbachia w*AlbB strain and uninfected cells generated by removing the bacterium through tetracycline treatment [35] were cultured at 28 °C in medium consisting of equal volumes of Mitsuhashi/Maramorosh (Bioconcept, Switzerland) and Schneider’s insect medium (Sigma, France), supplemented with 10% (*v*/v) of heat-inactivated fetal bovine serum (PAA, USA) and penicillin/streptomycin (50 U/50 μg/mL; Gibco, Invitrogen, France). Cells were continuously passaged in 25-cm^2^ flasks by scrapping and seeding a new flask with 1:5 of the cell suspension in 5 mL of fresh medium, every 4 days. The Chikungunya virus (CHIKV) 06.21 strain was isolated in C6/36 from newborn serum sample with neonatal encephalopathy during the outbreak in La Reunion Island [6]. Viral stocks were produced on C6/36 cells in 25-cm^2^ flasks, at Multiplicity Of Infection (MOI) of 0.01. After 3 days at 28 °C, supernatants from infected cells were recovered and virus titration was done using plaque assay on Vero E6 (green monkey kidney) cells [40]. The titer stock virus was estimated to 10^8^ plaque-forming units (PFU)/mL and stored in aliquots at −80 °C until used.

### Cell infection

To assess the impact of cell co-infection by *Wolbachia* and CHIKV, we compared four modalities of infection; cells uninfected, mono-infected by *w*AlbB or CHIKV and bi-infected, each with three independent biological replicates. The day prior infection, 5 × 10^6^ cells were transferred in 25-cm^2^ flask and allowed to attach for 18 h at 28 °C. Infection at MOI 0.1 with CHIKV 06.21 was performed in 0.5 mL new medium with 2% fetal bovine serum, using virus-free medium as control. After 1 h, 5 mL of fresh medium with 10% fetal bovine serum were added and incubation extended. Cells and supernatants were harvested at 24 and 120 h post-infection. For uninfected cells, we applied the same protocol but fetal bovine serum medium did not contain any virus particles. Blue trypan staining used for cell counting and light microscopy employed to monitor cell monolayers did not show apparent necrotic cells along the course of the experiment (not shown). At the two times (24 h and 120 h), cells were scrapped and pelleted by centrifugation and a fraction of these cells was conserved in 1.5 mL tube for genomic DNA and RNA isolations. Each cell pellet was washed once in 10 mL PBS 1× pH 7.4 (Gibco, Invitrogen, France) and then resuspended in lysis buffer composed of urea 7 M (Sigma, France), 2 M thiourea (Fluka, Sigma, France), 4% CHAPS (Sigma, France), 0.5% Triton ×100 (Sigma, France) and TBP 0.08 mM (Sigma, France) in distilled water (Gibco, Invitrogen, France); and incubated on ice for 30 min with regular vortexing. Cell lysates were stored at −80 °C until protein extractions.

### DNA and RNA isolation

Genomic DNA isolation was performed using *DNeasy blood and tissues Kit* (Qiagen, France) following manufacturer’s instructions. Cell pellets were resuspended in 180 μL of ATL lysis buffer and incubated for 2 h at 37 °C with 2 mg/mL lysozyme (Euromedex, France). Residual co-extracted RNA was eliminated by adding 100 mg/mL RNase A, for 2 min at room temperature, then isolated DNA was eluted in 30 μL of DNase-free water. To isolate total RNA, cell pellets were crushed in 350 μL RLT lysis buffer of *RNeasy Mini Kit* (Qiagen, France) using RNase-free piston pellet (Kontes, USA) and following manufacturer’s recommendations. Then RNA was eluted in 37 μL of RNase-free water and treated with DNase using the TURBO-DNA free kit (Ambion, USA) in 50 μL final volume following the manufacturer’s instructions. DNA and RNA were quantified using a UV-mc^2^ spectrophotometer and diluted to 5 ng/μL, then frozen at −20 °C (DNA) or −80 °C (RNA) until use.

### Quantitative analysis of *Wolbachia* (qPCR) and CHIKV (RT-qPCR)

To monitor the relative density of *Wolbachia* per cell, qPCR was performed using *Wolbachia* Surface Protein (*wsp*) gene for the bacterium and *actin* gene for the host cell. Standard curves were drawn on 10-fold serial dilutions from 1 × 10^8^ to 1 × 10^1^ copies/μL of the DNA plasmid pQuantAlb16S containing fragments of the two targeted genes [20, 41]. Amplification reaction was done in a total volume of 20 μL containing 10 ng of template DNA, 1× (10 μL) Fast-SYBR-Green Master Mix (Roche, Suisse), 200 mM of each *wsp* primers (5’AAGGAACCGAAGTTCATG3′ and 5’AGTTGTGAGTAAAGTCCC3’) and 300 mM each *actin* primers (5’GCAAACGTGGTATCCTGAC3’ and 5’GTCAGGAGAACTGGGTGCT3’). Amplification was performed on LC480 LightCycler (Roche, France) and consisted of 10 min at 95 °C, followed by 40 cycles of 15 s at 95 °C, 1 min at 65 °C, and a final elongation at 72 °C for 30 s. To quantify CHIKV RNA copy number, RT-qPCR was done on the envelope *E2* gene using a standard curve of 10-fold serial dilution of a synthetic CHIKV RNA transcript [29]. One-step RT-qPCR was performed using EXPRESS One-Step SYBR GreenER Kit (Invitrogen, France) in a volume of 20 μL containing 10 ng of RNA template, 1× (10 μL) EXPRESS SYBR GreenER SuperMix Universal, 200 nM of sense Chik/E2/9018/+ and anti-sense Chik/E2/9235/− primers [42] and 1× (0.5 μL) EXPRESS Superscript Mix. Amplification was performed on a LC480 LightCycler (Roche, France) and consisted of 15 min at 50 °C and by 95 °C for 2 min, followed by 40 cycles of 95 °C for 15 s and 63 °C for 1 min. All PCR reactions were done in triplicate. DNA and RNA extracted from C6/36 uninfected were used as negative control.

### Protein extraction, 2D–PAGE and densitometric gel analyses

To extract proteins, cell lysates were defreezed on ice and proteins were precipitated with 10% (*w*/*v*) trichloroacetic acid (Sigma, France) at 4 °C overnight. Proteins were pelleted by centrifugation at 14,000 g for 15 min at 4 °C and washed three times with glacial acetone (VWR Chemicals, France). Isoelectric focusing (IEF) was performed using the Protean IEF System (Biorad, France) according to the manufacturer’s instructions. The rehydration buffer contained 8 M urea (Sigma-Aldrich) and 4% (*w*/*v*) CHAPS (Sigma). IEF was performed with 11 cm no-linear strips, pH 3–10 (Biorad), using the Voltage Ramp protocol recommended by the manufacturer (100 V/30 min/rapid, 250 V/30 min/linear, 1000 V/30 min/linear, 7000 V/3 h/linear, and finally 32,000 V/h (pH 3–10 IPG)). The second dimension was carried out using the Criterion Dodeca system (Biorad). A minimum of four gels loaded with biological replicates was used for each condition. Criterion any kD TGX gels (Biorad) were run at 10 °C in Laemmli buffer [43] at 100 V for 2 h. Then the 2D–gels were stained with silver nitrate as previously described [44], scanned and analyzed using the software SameSpots v.4.5 (Non-linear Dynamics Progenesis, UK). An ANOVA test of the spot volumes was calculated to compare the different conditions. Variations in spot volumes with *p* < 0.02 and fold-change >2 were considered significant.

### Sample preparation and nanoLC-MS/MS analysis

Protein spots were destained in 60 mM potassium ferricyanide and 200 mM sodium thiosulfate mixed 1:1 until all brown color was removed. The spots were washed through successive incubations with water until all yellow color was removed and shrunk in acetonitrile (ACN) for 10 min. After ACN removal, gel pieces were dried at room temperature. Proteins were digested by incubating each gel slice with 10 ng/μL of trypsin (T6567, Sigma-Aldrich) in 40 mM NH4HCO3, 10% ACN, rehydrated at 4 °C for 10 min, and finally incubated overnight at 37 °C. The resulting peptides were extracted from the gel by three steps: a first incubation in 40 mM NH4HCO3, 10% ACN for 15 min at room temperature followed by two incubations in 47.5% ACN, 5% formic acid for 15 min at room temperature. The three collected extractions were pooled with the initial digestion supernatant, dried in a SpeedVac, and resuspended with 25 μL of 0.1% formic acid before nanoLC-MS/MS analysis. Online nanoLC-MS/MS analyses were performed using an Ultimate 3000 RSLC Nano-UPHLC system (Thermo Scientific, USA) coupled to a nanospray Q-Exactive hybrid quadruplole-Orbitrap mass spectrometer (Thermo Scientific, USA). Ten microliters of each peptide extract were loaded on a 300 μm ID × 5 mm PepMap C18 precolumn (Thermo Scientific, USA) at a flow rate of 20 μL/min. After 5 min desalting, peptides were online separated on a 75 μm ID × 25 cm C18 Acclaim PepMap® RSLC column (Thermo Scientific, USA) with a 4–40% linear gradient of solvent B (0.1% formic acid in 80% ACN) in 48 min. The separation flow rate was set at 300 nL/min. The mass spectrometer operated in positive ion mode at a 1.8 kV needle voltage. Data were acquired using Xcalibur 3.0 software in a data-dependent mode. MS scans (m/z 300–2000) were recorded at a resolution of *R* = 70,000 (@ m/z 200) and an AGC target of 1 × 10^6^ ions collected within 100 ms. Dynamic exclusion was set to 30 s and top 15 ions were selected from fragmentation in HCD mode. MS/MS scans with a target value of 1 × 10^5^ ions were collected with a maximum fill time of 120 ms and a resolution of *R* = 35,000. Additionally, only +2 and +3 charged ions were selected for fragmentation. Others settings were as follows: no sheath and no auxiliary gas flow, heated capillary temperature, 200 °C; normalized HCD collision energy of 25% and an isolation width of 3 m/z.

### Database search and results processing

Mascot, MS Amanda and Sequest algorithms through Proteome Discoverer 1.4 Software (Thermo Fisher Scientific Inc., USA) were used for protein identification in batch mode by searching against a merged database from http://www.uniprot.org/: *Aedes* (taxon identifier: [7158], 24,927 entries, release 2015_04) + *Wolbachia* (taxon identifier: [952], 24,150 entries, release 2015_04) + Chikungunya virus (taxon identifier: [37124], 2041 entries, release 2015_04) + Dengue virus (taxon identifier [12637], 13,782 entries, release 2015_04). Two missed enzyme cleavages were allowed. Mass tolerances in MS and MS/MS were set to 10 ppm and 0.02 Da. Oxidation of methionine, acetylation of lysine and deamination of asparagine and glutamine were searched as dynamic modifications. Carbamidomethylation on cysteine was searched as static modification. Peptide validation was performed using Target Decoy PSM Validator and only “high confidence” peptides were retained corresponding to a 1% False Positive Rate at peptide level. The mass spectrometry proteomics data have been deposited to the ProteomeXchange Consortium (http://proteomecentral.proteomexchange.org) via the PRIDE partner repository [45] with the dataset identifier PXD005091.

### Bioinformatics and statistical analysis

The continuous response variables (viral and bacterial titers) were log_10_-transformed. They were analyzed using a multifactorial linear model, with a normal error distribution and an identity link function that included the effect of the time and MOI as ordinal variables, treatment as discrete variable and their interactions. All the statistical analyses were performed using R environment (version 3.1.0). An annotation in GO term was carried out on the proteins identified using Blast2GO (3.2.7) then they were used to detect possible interaction networks using Cytoscape (3.3.0).

## Results and discussion

### *Wolbachia w*AlbB affects CHIKV *in cellulo*

**Fig. 1:**
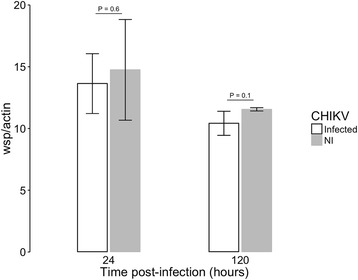
Density of *w*AlbB in C6/36 cells during CHIKV infection. Ratio of *Wolbachia wsp* copies per host *actin* copies during CHIKV infection at MOI 0.1, measured by qPCR on genomic DNA. Error bars represent the standard deviation of the mean of three independent samples

**Fig. 2:**
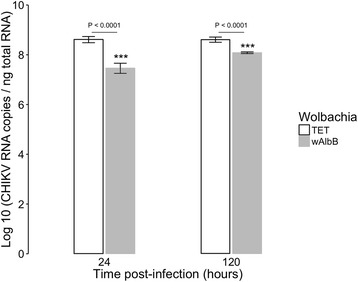
Kinetics of CHIKV RNA titer upon *Wolbachia* infection. Effect of *Wolbachia* on CHIKV RNA titer at MOI 0.1 measured by RT-qPCR on total cellular RNA in presence of *w*AlbB or in cells cured of the bacteria by tetracycline treatment (TET). Error bars represent the standard deviation of the mean of three independent samples

As our previous study of the C6/36 infected with *w*AlbB showed that presence of the bacterium decreased the viral titer compared to uninfected cells [35], we measured *w*AlbB and CHIKV densities at 24 and 120 h post infection (p.i.) using qPCR and RT-qPCR, respectively. The density of *Wolbachia* was about 12 *wsp* gene/*actin* ratio (Fig. 1). The percentage of *Wolbachia*-infected cells ranged from 60 to 70% (not shown) as determined by fluorescent in situ hybridization published protocol [35]. The CHIK RNA copy number was estimated between 10^7^ to 10^9^ per ng of total RNA (Fig. 2). Both *Wolbachia*-infected and uninfected cells produce infectious viral particles without visible cytopathic effect (not shown). This was expected as *Aedes* cells are permissive to many arboviruses, including CHIKV, that are found non pathogenic to mosquitoes [46, 47]. This is why the C6/36 cell line is extensively used to propagate viruses [48].

Statistical analyzes demonstrated that the density of *Wolbachia* was not affected by the presence of the CHIKV, and was marginally affected upon time (*P* = 0.05262) (Fig. 1). As expected, the viral titer was significantly reduced in the presence of *Wolbachia* (*P* < 2.2e-16), without reaching complete inhibition. The inhibitory effect decreased with time, being lower at a late time (*P* = 0.0007825) (Fig. 2). It has been reported that viral inhibition by *Wolbachia* is density-dependent [28, 37]. At the two time points tested here the *Wolbachia* density remained stable, around 12 bacteria per cell, and the level of CHIKV inhibition was similar to previous studies [35]. The chronic *Wolbachia* infection and the permissiveness to viruses make the C6/36 cell line an interesting model for exploratory functional studies. One unfavorable point of this cell line is the lack of siRNA pathway [49], a primary immune response against viral infection in mosquitoes. However, it has been shown that insects can mobilize other RNA interference pathways to control viral replication. For instance, *Aedes aegypti* induces miRNA and specific piRNA pathways to control the replication of DENV [50–52]. Similarly, *Wolbachia* could have an effect on synthesis of small RNAs [53, 54]. Therefore, this cellular model seems suitable for the study of induced host-cell responses following mono- or bi-partite infection by *Wolbachia* and/or CHIKV as well as the CHIKV replication cycle.

### Differential cell proteome profiles upon microbial infection

**Fig. 3:**
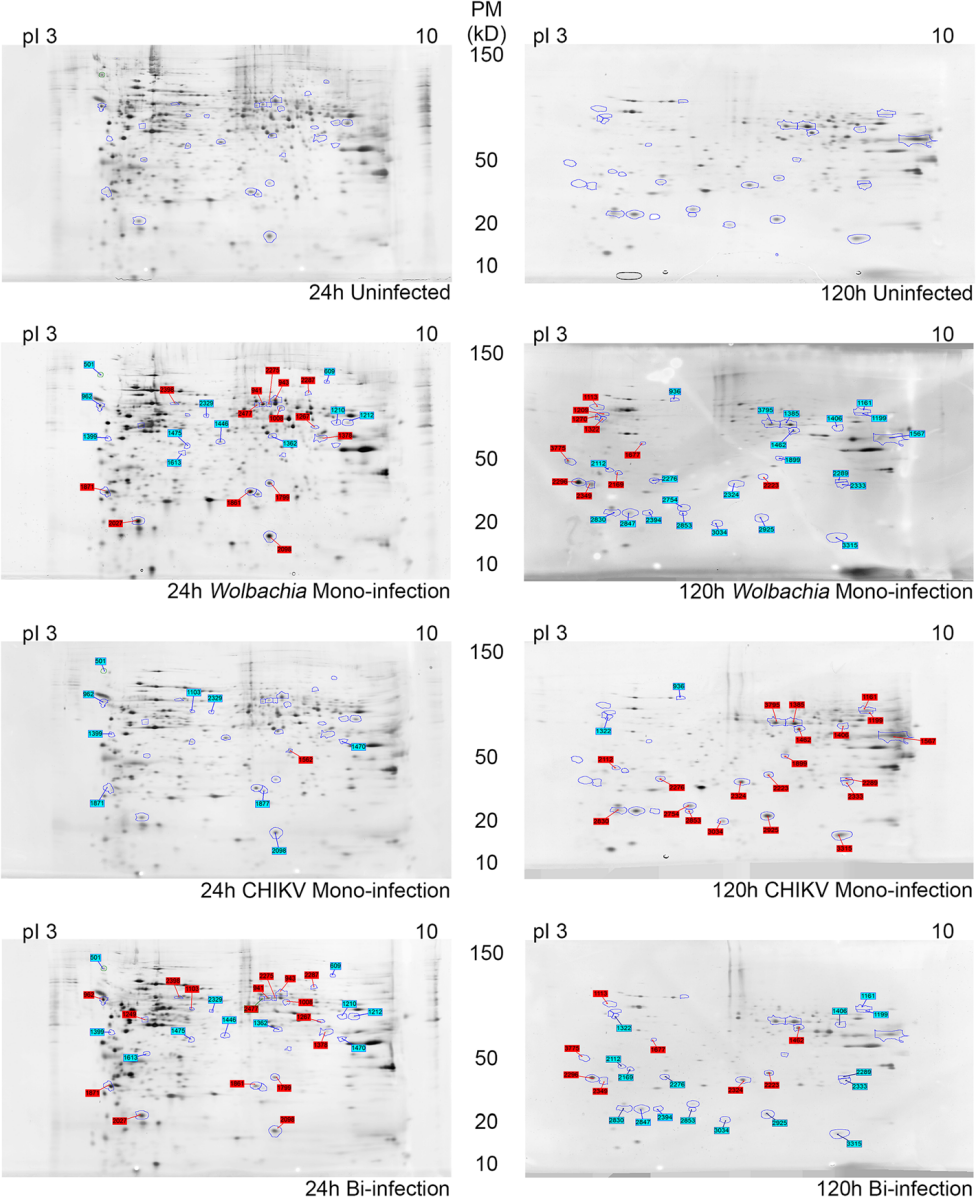
Two D electrophoresis Gels of C6/36 extracts showing spots modulated after analysis. Protein spots differentially expressed are indicated by numbers, in blue for down-regulation and red for up-regulation as normalized in respect to uninfected cells. The pI range (3 to 10) is indicated on top of the gels, and molecular weight beside the gels

For the two time points (24 h and 120 h p.i.) and the four modalities (uninfected, mono-infected by either *Wolbachia* or CHIKV and bi-infected by both microbes), three independent biological replicates were performed. Total proteins were extracted and similar amounts (approximately 150 μg, estimated on a 1D gel) were used for 2DE. For each modality and each replicate, a minimum of 4 and a maximum of 5 gels were used. Typical 2D gels with spots obtained are illustrated in Fig. 3. The global gel analysis using the ProGenesis SameSpots software enabled detection of 906 spots at 24 h and 901 spots at 120 h p.i. ANOVA analysis allowed identifying 58 spots at 24 h and 32 spots at 120 h p.i that were statistically different (*p* < 0.02 and fold change >2) in comparison to uninfected cells. As many of the spots identified at early time point were linked to *Wolbachia* infection alone, only 30 of the 58 spots were selected for mass spectrometry sequencing, including all 32 spots observed at the late time point.

A protein was considered present in a spot when a minimum of two different peptides were identified by mass spectrometry (Additional file 1: Table S1). Consequently, a total of 495 unique proteins were identified from 948 sequences at 24 h p.i., whereas 105 unique proteins were found among 168 sequences at 120 h p.i. The elevated number of identified sequences in the analysis can be explained by two major reasons; (i) a high number of proximate proteins that have possibly been subjected to post-translational modifications and (ii) protein fragmentation during experimentation that resulted in modified migration patterns. All peptide sequences and observed fold changes are described on the Additional file 1: Table S1. By combining the protein level in each time point and the modality of infection, a total of four major profiles were defined, including monoinfection, dominance, cumulative and interference (Table 1). Accordingly, in the monoinfection profile each microbial partner tends to affect a particular protein or a group of host proteins. The dominant profile indicates a major impact of one microbial partner on the host protein synthesis (up or down) whereas the other microbial partner showed an opposite profile. The cumulative effect means that the two microbial partners displayed a synergic effect on protein synthesis. Lastly, the interference profile indicates that each microbial partner induces a specific protein pattern but the co-infection displays a totally new trend.

**Table 1:** Profiling of protein accumulation

Profile		Profile name	Time	*w*AlbB	CHIKV	Bi-infection	Number of spots	Network
Mono Infection	*Wolbachia*	W_Up_1	24 h	Up	ø	Up	13	
		W_Up_2	120 h	Up	ø	Up	5	
		W_Down_1	24 h	Down	ø	Down	7	
		W_Down_2	120 h	Down	ø	Down	2	
	CHIKV	V_Down	24 h	ø	Down	Down	1	
Dominance	*Wolbachia* dominance	W_DOM_1	24 h	ø	Up	ø	1	Fig. 5a
		W_DOM_2	24 h	Up	Down	Up	2	Fig. 5b
		W_DOM_3	24 h	ø	Down	ø	1	Fig. 5c
		W_DOM_4	120 h	Down	Up	Down	12	Fig. 5d
	CHIKV Dominance	V_DOM_1	120 h	Up	ø	ø	2	Not Shown
		V_DOM_2	120 h	Up	Down	Down	1	Fig. 6a
		V_DOM_3	120 h	Down	Up	Up	2	Fig. 6b
Cumulative		CUMUL_1	24 h	Down	Down	Down	3	Fig. 7a
		CUMUL_2	120 h	Up	Up	Up	1	Fig. 7b
Interference		INT_1	24 h	Down	Down	Up	1	Fig. 8d
		INT_2	24 h	ø	Down	Up	1	Fig. 8a
		INT_3	120 h	Down	Up	ø	5	Fig. 8b
		INT_4	120 h	Down	Down	ø	1	Fig. 8c
		INT_5	120 h	Up	ø	Down	1	Fig. 8e

All profiles were normalized with respect to uninfected modality. Effective observed fold changes are reported on Additional file 1: Table S1. In comparison to uninfected C6/36 cells: Up: A positive difference on protein synthesis has been observed; Down: A negative difference on proteins synthesis has been observed; ø: No difference has been observed

The 2DE combined with mass spectrometry sequencing did not allow quantification of the level of protein accumulation per spot, and one spot can contain several proteins, consequently it was not possible to identify which protein was involved in the variation observed. In addition, the presence of many identical proteins in several spots simultaneously makes the analysis complex. Therefore we proceeded by annotating proteins in GO terms that were used to construct interacting networks for each protein profile. This procedure allowed comparison of functions shared by all modalities with those belonging specifically to each partner.

### *Wolbachia* infection has a greater effect on cell functions than CHIKV

**Fig. 4:**
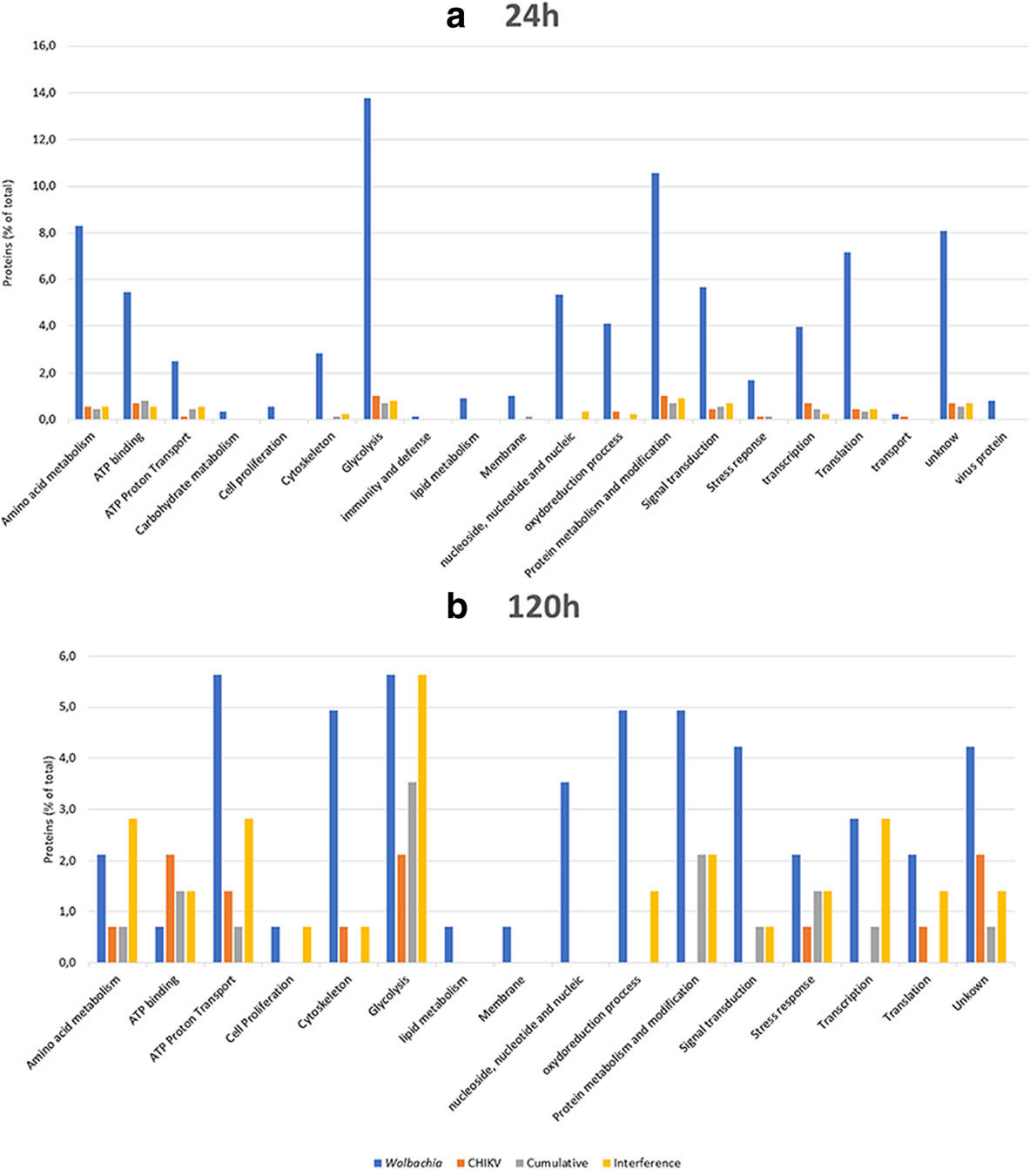
Functional categorization of proteins found in differentially regulated spots of C6/36 cells under different infection modalities. **a** At 24 h post infection, (**b**) at 120 h post infection by CHIKV. Blue bars, functions impacted by *Wolbachia* (Monoinfection and dominance profile); red bars, functions impacted by CHIKV (Monoinfection and dominance profile); gray bars, functions impacted by cumulative effect; yellow bars, functions impacted by interference effect. The results are expressed in % values of total differentially accumulated proteins by cell functions (*p* < 0.02; 2-fold change)

Among the 89 spots detected, 77% were specifically synthesized in the presence of *Wolbachia* in both mono-infection (53 spots) and dominance (16 spots) profiles (Table 1). Since the aim of this study was to characterize the impact of coinfection rather than monoinfection, we have chosen to sequence only 27 out of 53 spots, that were selected on the basis of a particular fold change as indicated above. Results of sequencing showed that spots linked to *Wolbachia* in both mono-infection and dominance profiles contained proteins involved in many cellular functions, including processes related to metabolism for acquisition of resources from the host, regulation of anti-oxidation and cellular functional machinery (transcription and translation), as well as active transport and cellular structures (Fig. 4). These proteins were present at the two times at relatively high percentage (64.5% at early time and 35% at late time) of the total proteins, and some of them have been already described in literature as being upregulated by *Wolbachia* [39]*.* One example is the Glutathione S-transferase (A0A023EL34) for the regulation of anti-oxidation process [38], which is abundant at early time in the presence of the bacterium. The large number of proteins mobilized in the presence of *Wolbachia* indicates a strong relationship between the two partners.

In contrast, the presence of CHIKV alone has only limited effect in comparison to uninfected cells. Few differential spots containing proteins at a very low percentage (<5%) were detected, with tendency to be down-regulated. The majority of the proteins detected were related to the ATP transport and binding, glycolysis, cytoskeleton and stress responses (Fig. 4). For instance, many proteins associated with ATP consumption were significantly reduced in the presence of CHIKV. Moreover, we observed a decrease in expression of the gene encoding A0A023END7 LSD2 (Lipid Storage Droplet-2), suggesting that CHIKV blocks lipid storage, potentially making them available incorporation into the viral envelope. This phenomenon has already been shown in *Ae. aegypti* mosquitoes infected by either dengue [55] or chikungunya viruses [14, 15]. Another protein A0A023EQG9 negatively impacted encoded a kinase for double-stranded RNA necessary to the establishment of RISC complex in RNA interference phenomenon. Knowing that the C6/36 cell line has a non-functional siRNA mechanism [49], inhibition of the miRNA pathway is consistent with a viral mechanism to escape cellular defenses.

**Fig. 5:**
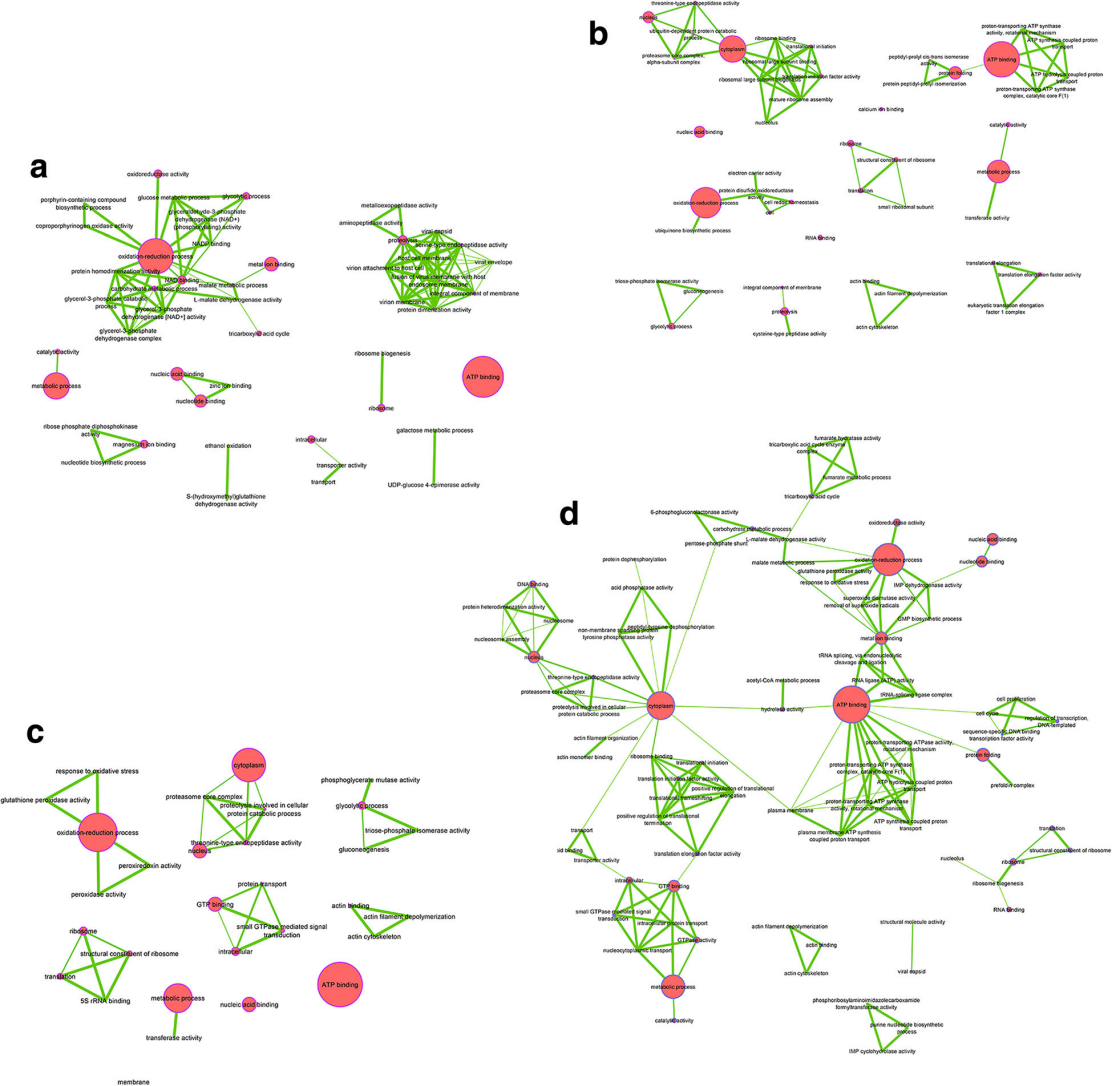
*Wolbachia* Dominance function network. **a** W_DOM_1, (**b**) V_DOM_2, (**c**) W_DOM_3, (**d**) W_DOM_4. Network of functions impacted by *Wolbachia* infection, A, B and C at 24 h post infection by CHIKV and D at 120 h post infection. Largest nodes mean that a greater number of proteins was related to this function

In dominance profiles, *Wolbachia* exhibited different protein trends in respect to virus, from a neutral level (W_DOM_1 and W_DOM_3), an increased (W_DOM_2) or repressed (W_DOM_4) synthesis (Fig. 5). The W_DOM_1 profile reduced the CHIKV structural polyprotein V5UMV1 at 24 h post infection (hpi)., whereas the W_DOM_4 profile targeted specifically the viral capsid protein (A0A059VQ68) at 120 hpi (Table 1). These results are in agreement with the *Wolbachia* blocking phenotype observed recently for CHIKV in C6/36 cells [35]. Viral blocking is therefore explained by inhibition of *Wolbachia* cellular proteolysis machinery, thus limiting the maturation of virion-associated protein structures and reducing viral replication. Overall, this effect appeared more diverse at early stages post-infection, but of greater magnitude at later times (Table 1). At 24 hpi *Wolbachia* tends to sustain necessary cellular processes, such as oxidizing processes including glutathione peroxidase activity, translation and transcription (Fig. 5). Whereas, this is not the case at 120 hpi, when the bacterium limits processes that will be exploited by the virus, including oxidative stress, transportation and translation.

**Fig. 6:**
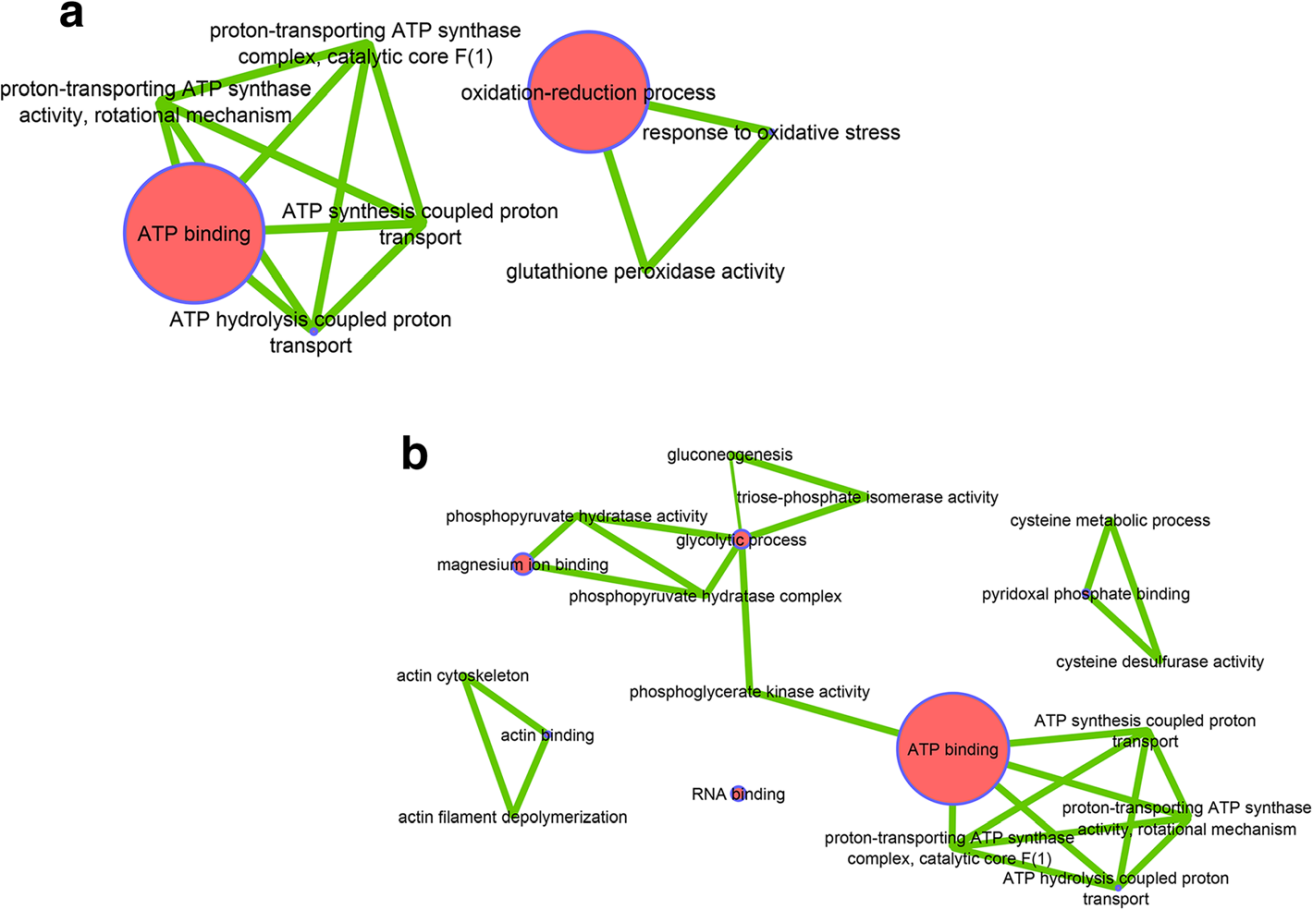
CHIKV Dominance function network. **a** V_DOM_2, (**b**) V_DOM_3. Network of functions impacted by CHIKV infection. Largest nodes mean that a greater number of proteins was related to this function

The viral dominance profile occurred at 120 hpi, when the virus had established chronic and dense infection (Fig. 6). The ATP synthase subunit beta (A0A023ETB9), involved in active trans-membrane ion transport, appeared negatively regulated as well as Glutathione peroxidase (Q16N54), albeit to a lesser extent. In contrast, some structural proteins such as actin (Q0Z987) and those related to heat shock (A0A023EWK8) were over-synthesized, suggesting a role in the production of virions [17]. The presence of ATP synthase subunit beta in both up and down-regulated profiles suggests several isoforms of this protein that *Wolbachia* modulates by regulating post-translational modifications.

### Proteome trends during *Wolbachia* and CHIKV coinfection

**Fig. 7:**
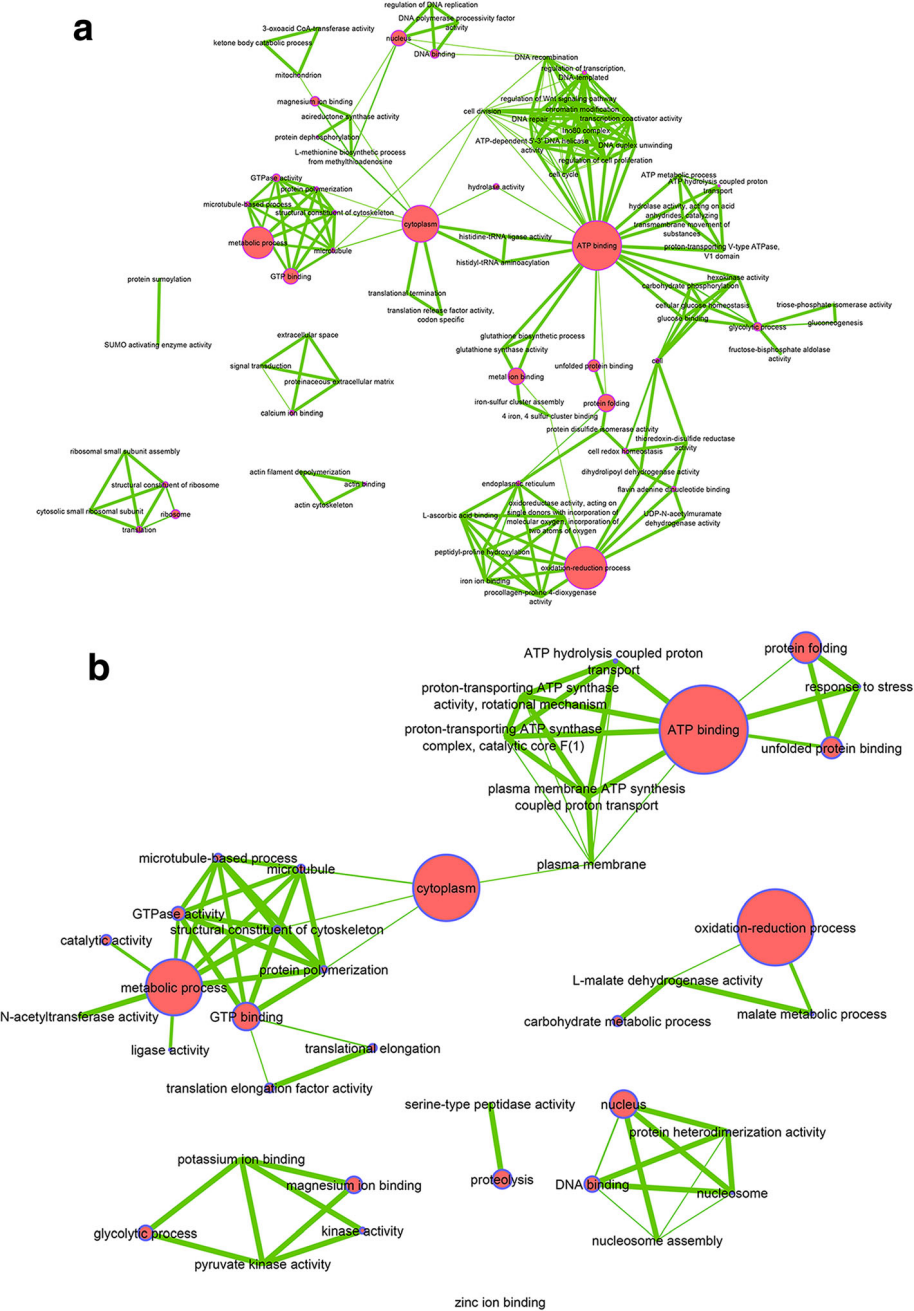
Cumulative effect function network. **a** CUMUL_1, (**b**) CUMUL_2. Network of functions impacted by *Wolbachia* and CHIKV infection, A at 24 h post infection by CHIKV and B at 120 h post infection. Largest nodes mean that a greater number of proteins was related to this function

Two different profiles emerged from bacterial and viral coinfection. The first was a cumulative profile in which a synergistic negative effect on protein synthesis was observed (Fig. 7). The processes observed to be affected by bi-infection were those already identified during infection by bacteria and viruses [14, 15, 17]. These proteins all act to maintain cell integrity and are associated with either down-regulation early post infection or up-regulation late post infection.

**Fig. 8:**
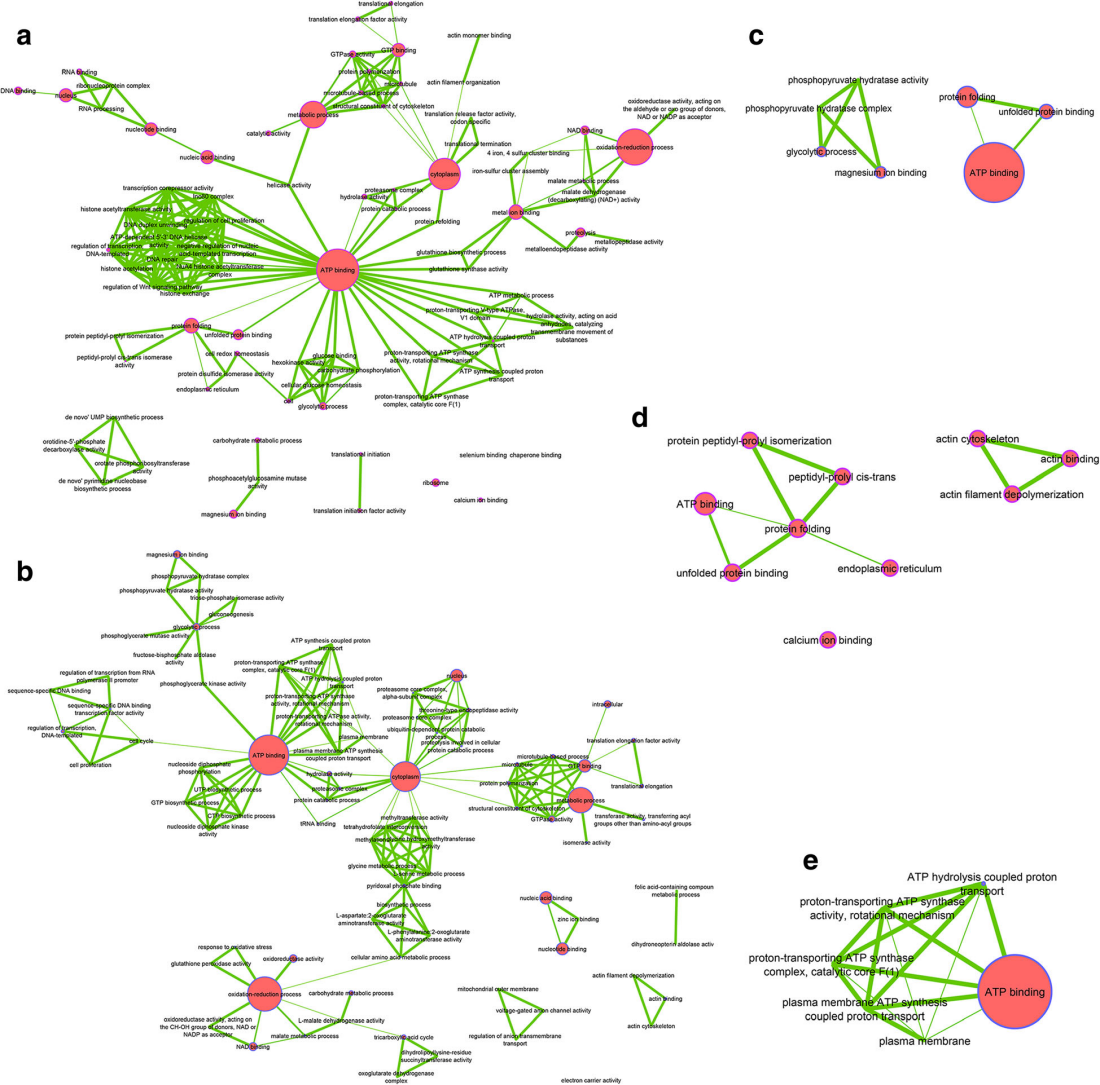
Interference effect function network. **a** INT_1, (**b**) INT_2, (**c**) INT_3, (**d**) INT_4, (**e**) INT_5. Network of functions impacted by *Wolbachia* and CHIKV infection, A and B at 24 h post infection by CHIKV and C, D and E at 120 h post infection. Largest nodes mean that a greater number of proteins was related to this function

The second pattern was an interference profile (Fig. 8). At 24 hpi, interference seemed to be directed against CHIKV and in favour of *Wolbachia*. Indeed, despite *Wolbachia* neutral (INT_2) or negative (INT_1) effects, cellular processes that were found to be up-regulated were those that may be of benefit to the bacterium, including cell development processes, transcription, translation and various metabolic pathways. At 120 hpi the INT_3 profile showed establishment of a balance between *Wolbachia,* which decreases metabolic processes, and the virus who in turn activated them for its own benefit. This sum of effect allows maintaining these processes at a steady-state level in cell. INT_4 profile was essentially related to structural proteins that were inhibited by each microbial partner, but during bi-infection where these proteins were not down-regulated. INT_5 profile identified ATP synthase subunit beta of *Wolbachia* (H0U0S7) that was inhibited by the virus. This later profile highlights a particular pattern where the presence of virus inhibited bacterial proteins through the blocking access to resources, thus limiting the potential of the bacterium to affect the virus.

When comparing the peptides detected in this study with those already described in mono-infection models using mass spectrometry approaches, some common proteins were identified. These include enolase (A0A023ETA6), which was found to stimulate transcription of the Sendai virus genome [56], and upregulated in the interference profile (INT_3). If CHIKV seems to enhance the enolase synthesis, as already shown by Lee et al. [17], *Wolbachia* tends to reduce its production. Consequently, in the bi-infection status, this conflictive pattern appears unfavorable to CHIKV replication. Among proteins involved in glycolysis and metabolism, one promising candidate is the disulfide isomerase protein (A0A023EP23) which has been shown to be modulated by CHIKV according to the infected organs [14, 15] and the duration of infection [17]. In our study, this protein is modulated by *Wolbachia* (W_Up_1), affecting the early CHIKV replication. Similarly, some chaperonins such as the putative calreticulin-like 2 (A0A023EQL3), chaperonin 60 kDa (A0A023EV59), heat shock cognate 70 (Q1HQZ5), alpha and beta tubulin 1 (A0A023ERN1 and A0A023ESE6) have been described to be modulated during CHIKV infection [14, 15, 17], and for which we found to be impacted by bi-infection status. These observations are also operating in glycolysis with for instance triosephosphate isomerase (A0A023EIM8) shown to be important in energy input necessary for viral replication. Indeed, at early time, this protein is overexpressed in *Wolbachia*-infected cells, inducing a favorable environment for CHIKV. In contrast, at latter time, *Wolbachia* seems to reduce the expression of triosephosphate isomerase while CHIKV tends to increase its activity (V_DOM_3 profile), suggesting the importance of such protein in this tripartite interaction.

## Conclusions

This study highlights complex processes that occur during arbovirus infection of mosquito cells in symbiosis with *Wolbachia*. Even though these findings were obtained using a cellular model, the observed trends pave the way for future research into the in vivo characteristics of tripartite interaction. In our experimental conditions, the combination of 2DE and nanoLC-MS/MS revealed a balance in protein synthesis mostly in favor of *Wolbachia*, which may explain the simultaneous inhibition of viral replication that we observed using RT-qPCR. At early times post infection, the presence of *Wolbachia* greatly influences many cellular processes related to management of anti-oxidant activity, protein production, various metabolic pathways linked to the provisioning of resources; likely impacting CHIKV replication. Under such conditions, CHIKV faces a hostile environment for replication and appears to counterbalance this negative impact by blocking some key cellular pathway, including the inhibition of transcription, translation and locking of an miRNA pathway.

At later times post infection, the proteome is clearly altered, and CHIKV activity seems to have taken control of some cellular functions. Consequently, the virus seems to limit the impact of *Wolbachia* on its replication cycle by hoarding the majority of resources, and even blocking *Wolbachia*’s access to these resources. This shift partially explains the increased viral titer that is observed at later periods post-infection. Even if *Wolbachia* no longer controls some of these cellular processes, its presence limits the effect of CHIKV infection on certain cellular functions, thus modulating its replication, particularly early after the infection process. This cellular level interference could explain phenotypes observed in *Ae. albopictus* in vivo, where *Wolbachia* limits transmission of dengue virus by reducing the viral titer in salivary glands [27].

Several studies have shown that *Wolbachia* can modulate the expression of genes involved in immunity that affect arbovirus infection, suggesting that interference acts by pre-immunization of the host [26, 28, 34]. Strikingly, we do not observe significant modulation of proteins related to immune response upon CHIKV-inhibition by *Wolbachia.* Even though C6/36 lacks functional siRNA pathways, other immune response mechanisms could have been mobilized. The fact that we did not identify proteins involved in immunity might suggest that other cellular processes can lead to the antiviral profile, corroborating results obtained from other cellular models. For example, in *Ae. albopictus* Aa23 cells infected with either *w*AlbB, *w*Mel or *w*MelPop, whose density varied from 2.5 to 38 bacteria per host cell, no changes were observed in innate immunity related functions [57]. Recently, an elegant work demonstrated that *Wolbachia* could inhibit viral replication at early stages post infection by affecting RNA translation or transcription, suggesting a likely direct effect [58]. Together these cellular models revealed alternative mechanisms to immunity in *Wolbachia*-based viral inhibition that need further investigations. An interesting perspective could be the extension of proteome profiling to mosquito organs as well as testing other *Ae. albopictus*-transmitted arboviruses, such as Dengue and Zika, with emphasis on functions revealed in this study.

## Additional file

Additional file 1: Table S1. List of spots and fold change with sequences of peptides. (XLSX 7247 kb)

## Abbreviations

ATP: Adenine triphosphate
CHAPS: 3-[(3-cholamidopropyl) dimethylammonio]-1-propanesulfonate
CHIKV: Chikungunya virus
FBS: Foetal bovine serum
GO: Gene ontology
IEF: Isoelectric focusing
IPG: Immobilized pH gradient
LC-MS/MS: Liquid chromatography coupled to tandem mass spectrometry
MOI: Multiplicity of infection
p.i.: Post-infection
PBS: Phosphate buffered saline
TBP: Tributylphosphin
TCA: Trichloroacetic acid
TET: Tetracycline
*w*Alb_B: *Wolbachia B*
